# Not Just an Accident: A Case of Insulinoma

**DOI:** 10.7759/cureus.48514

**Published:** 2023-11-08

**Authors:** Asanka B Ekanayake, Harrison Stubbs, Dominique Broutin, Olga Karasik, Mustafa Kinaan

**Affiliations:** 1 Internal Medicine, HCA Florida Osceola Hospital, Kissimmee, USA; 2 Internal Medicine, University of Central Florida College of Medicine, Orlando, USA; 3 Endocrinology, HCA Florida Osceola Hospital, Kissimmee, USA

**Keywords:** pancreatic insulinoma, severe hypoglycemia, c-peptide, proinsulin, high serum insulin and c-peptide

## Abstract

Insulinomas are a rare cause of recurrent hypoglycemia in non-diabetic patients. Diagnosis requires hypoglycemia (plasma glucose <50 mg/dL), neuroglycopenic symptoms, and prompt relief of symptoms following the administration of glucose, known as Whipple’s triad. The gold standard diagnostic tests are measuring insulin, C-peptide, and glucose during a 72-hour fast. In the preoperative period and in patients with unresectable or metastatic tumors, medical management with diazoxide and octreotide can be considered for recurrent hypoglycemia.

We present a case of insulinoma in a 37-year-old woman who initially presented after a seizure-related motor vehicle accident. Upon admission, her initial glucose level was 32 mg/dL, indicating a likely hypoglycemic seizure. During her hospitalization, she had recurrent episodes of fasting and postprandial hypoglycemia, ranging from 32-70 mg/dL. She exhibited the characteristics of Whipple's triad when values dropped below 50 mg/dL. These episodes necessitated continuous infusions of 10% dextrose. Tests for insulin autoantibodies, sulfonylurea screens, and thyroid function yielded unremarkable results.

A 72-hour fasting test was initiated to investigate potential endogenous causes of excessive insulin production. Laboratory results from a venous glucose level of 46 mg/dL indicated a notable rise in C peptide and insulin levels, alongside beta hydroxybutyrate suppression, all of which fulfilled the diagnostic criteria for insulinoma. An abdominal magnetic resonance imaging (MRI) unveiled a 1.3 cm mass in the pancreatic tail.

This case emphasizes the importance of employing a focused approach when evaluating non-diabetic individuals displaying hypoglycemia with positive Whipple's triad. This targeted method not only enables early detection of this rare condition but also assists in eliminating other common causes of recurrent hypoglycemia in non-diabetic individuals. Moreover, in addition to this diagnosis being rare, it is important to note that patients with insulinomas typically do not exhibit a glucose level low enough to induce seizures during their initial presentation.

## Introduction

Insulinomas, a subset of uncommon neuroendocrine tumors, occur at a yearly rate of one to three cases per million individuals within the general population [1]. Typically benign and sporadic, these tumors are generally small (<2 cm in diameter). Upon presentation, the Whipple's triad is often present, which includes hypoglycemia, neuroglycopenic symptoms, and relief of symptoms upon glucose administration. Neuroglycopenic symptoms can range from dizziness, confusion, and visual disturbances to seizures and even coma. The rarity of insulinomas and their nonspecific symptoms often lead to a delayed or missed diagnosis. About 10% of insulinomas occur as part of multiple endocrine neoplasia type 1 (MEN1) syndrome and are multiple [1-5]. A combination of clinical presentation, laboratory tests, and imaging studies are used for diagnosis and lesion localization. Surgical resection is the definitive treatment [6]. In this report, we present a patient who initially presented with an insulinoma-induced seizure-related motor vehicle accident [7]. The case was originally presented at the 2023 American Association of Clinical Endocrinology (AACE) annual meeting on May 4-6, 2023.

## Case presentation

Following a motor vehicle accident, a 37-year-old woman with a history of gestational diabetes was hospitalized for an evaluation of recurrent hypoglycemia. Initially, she presented as loss of consciousness resulting from a witnessed seizure, that improved with the administration of 5% dextrose (D5W) at the scene of the accident. She reported complete amnesia of the episode and at initial presentation her glucose was 32 mg/dL. Examinations of the neurological, cardiovascular, and respiratory systems revealed no abnormalities. On arrival at the emergency department (ED), the patient was not in status epilepticus and her Glasgow Coma Scale was 12 with normal vital signs. Psychomotor activity was normal; perception and cognitive functions were normal.

Throughout her hospitalization, the patient consistently experienced recurrent episodes of hypoglycemia, both fasting and postprandial. During these episodes, glucose levels would range from 32 to 70 mg/dL. These episodes became symptomatic when her glucose dropped below 50 mg/dL, accompanied by Whipple’s triad criteria, necessitating the administration of continuous 10% dextrose solution infusions.

Although she was diagnosed with gestational diabetes during her last pregnancy three years prior, she reported no subsequent diagnosis of diabetes. The patient also reported no prior symptoms of hypoglycemia such as weakness, diaphoresis, pallor, tremors, confusion, paresthesias, or visual blurring in the past year. Moreover, she confirmed the absence of any glycopenic symptoms prior to this hospitalization and asserted that she wasn't using any medications or supplements while at home.

The patient's insulin autoantibodies and sulfonylurea screen tests returned undetectable, while her thyroid labs were normal. Evaluation for adrenal insufficiency with cosyntropin stimulation test was normal. To investigate the possibility of endogenous insulin overproduction, a 72-hour fasting test was initiated. A venous glucose measurement of 46 mg/dL yielded noteworthy increases in C-peptide and insulin concentrations. Additionally, there was a notable suppression of beta hydroxybutyrate levels, meeting the diagnostic criteria for insulinoma (Table 1).

**Table 1:** Patient's diagnostic lab values versus expected diagnostic values in insulinoma.

	Patient's labs	Expected Diagnostic Values in Insulinoma
Glucose (mg/dL)	46	< 55
C-Peptide (nmol/L)	1.35	﻿> 0.2
Proinsulin (pmol/L)	193.9	﻿> 5
Insulin (microU/mL)	13.2	﻿> 3
Beta hydroxybutyrate [mmol/L)	0.086	< 2.7
Insulin antibody	Negative	Negative
Sulfonylurea	Negative	Negative

**Figure 1:**
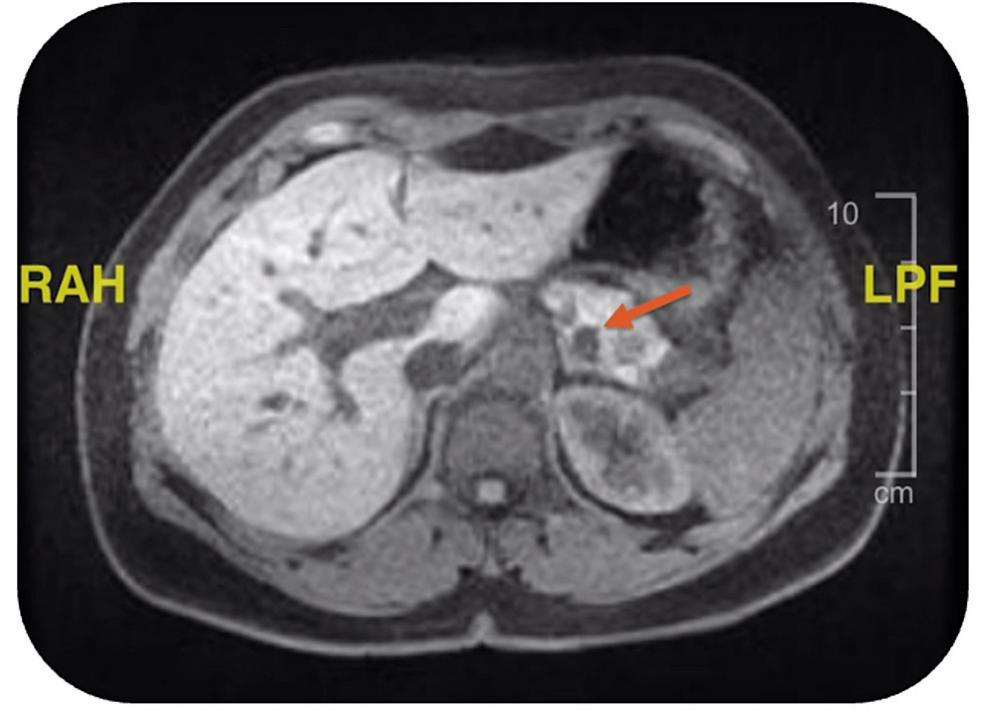
Abdominal MRI T1 pre-contrast imaging demonstrating 13 mm focal area of lower signal intensity in the tail of the pancreas (orange arrow). LPF = Low Pass Filter, RAH = Radiological Axis System

**Figure 2:**
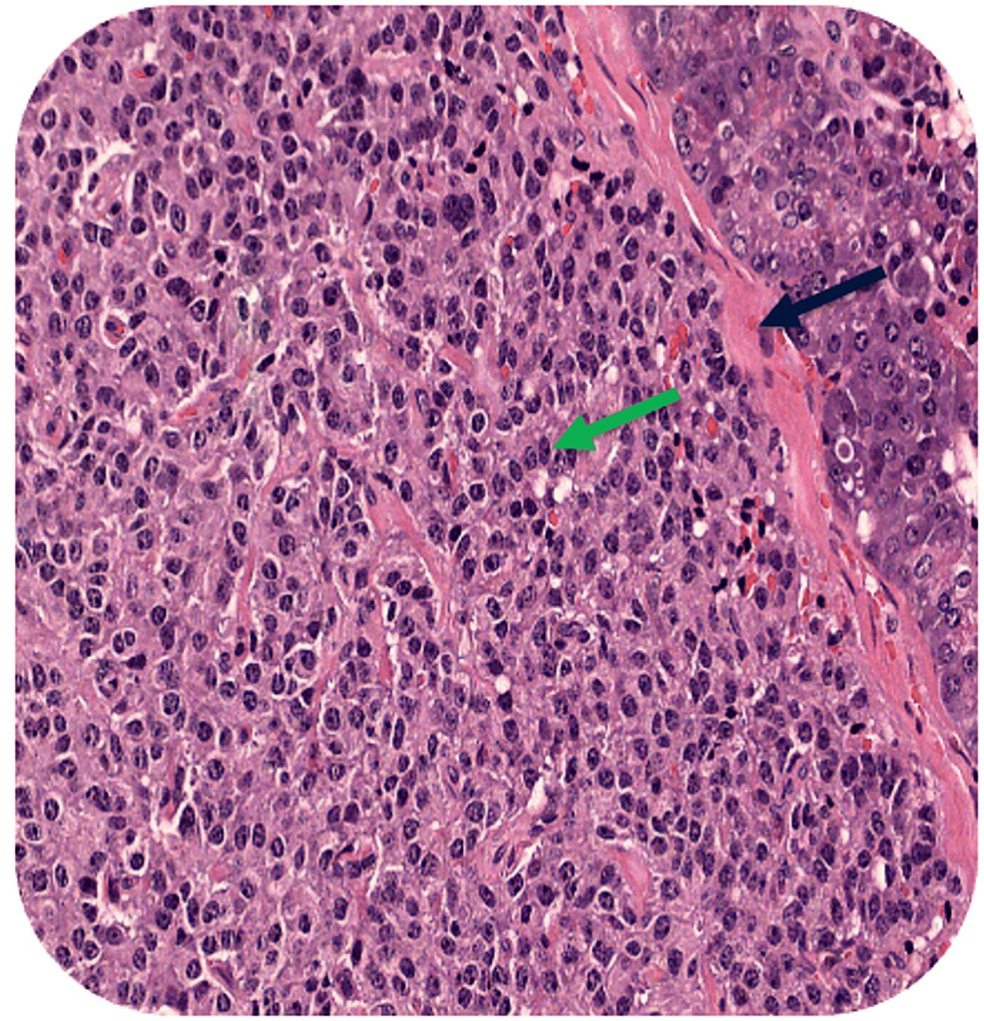
Monotonous bland cells in chords. Synaptophysin and chromogranin immunohistochemical stain strongly positive within the tumor cells (green arrow). This immunohistochemical profile is compatible with well-differentiated neuroendocrine tumor, grade 2. Amyloid deposition, characteristic of an insulinoma (black arrow)

An abdominal magnetic resonance imaging (MRI) with contrast revealed a 1.3 cm mass located at the tail of the pancreas (Figure 1). Subsequently, the patient underwent a laparoscopic distal pancreatectomy, and the tumor underwent pathological assessment, uncovering a well-differentiated neuroendocrine tumor (Figure 2). The tumor cells exhibited strong positive staining for synaptophysin and chromogranin on immunohistochemistry. The Ki-67 immunohistochemical stain demonstrated a proliferation index of 5%. These findings indicated a well-differentiated neuroendocrine tumor of grade 2. All margins were found to be free of tumor.

During a brain MRI, an incidental pituitary microadenoma was also discovered, which raised concerns about the presence MEN1 syndrome. Nonetheless, the patient displayed no biochemically detectable signs of parathyroid or pituitary disorder, and genetic testing yielded negative results for MEN1 syndrome. Following surgery, the patient had a positive response with no recurrence of hypoglycemia and was safely discharged home.

## Discussion

Insulinomas represent an uncommon source of recurrent hypoglycemia among individuals without diabetes, with an annual incidence ranging from one to three cases per million. The majority, over 90%, of insulinomas are benign. These growths tend to manifest more frequently in patients who are middle-aged, female, and those with a higher body mass index (BMI) [1,2,4,5,8]. Classically, insulinomas are said to follow the “rule of 10” stating that 10% are multiple, 10% are malignant, 10% are associated with MEN1, and 10% are ectopic [2]. Approximately 30% arise in each of the body, tail, and head of the pancreas [9]. 

Patients diagnosed with insulinoma typically exhibit preceding symptoms of hypoglycemia that arise from neuroglycopenia and heightened catecholamine discharge [10]. However, the diagnosis can often be missed or delayed due to the incomplete functional activity. Reduced hypoglycemic symptoms and negative laboratory findings are frequently misdiagnosed as psychiatric, cardiac, or neurological conditions [11]. Additionally, the rarity of the condition and the complex diagnostic workup can lead to delays in diagnosis. Based on a 60-year longitudinal study, the majority of individuals diagnosed with insulinoma experienced neuroglycopenic symptoms within 1.5 years before the diagnosis [12]. Interestingly, our patient denied any significant symptoms prior to her motor vehicle accident. That being said, while hospitalized she became symptomatic when her blood glucose dropped below 50 mg/dL, suggesting that her body may have adapted to normal functioning at lower glucose levels.

**Figure 3:**
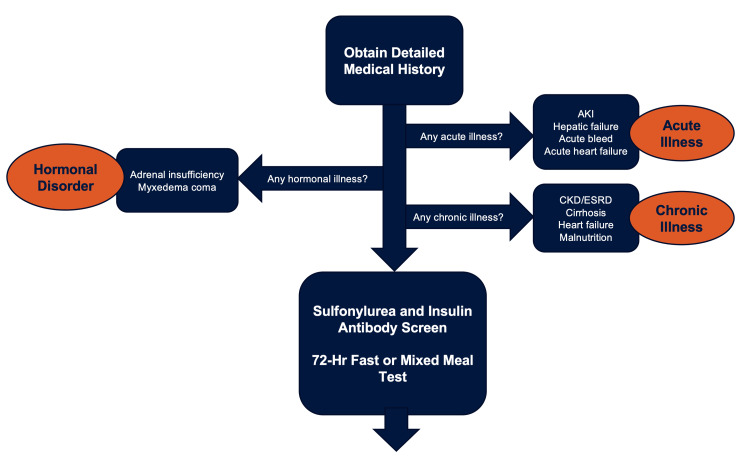
Algorithm for evaluation of recurrent hypoglycemia in non-diabetic patients. AKI: Acute Kidney Injury, CKD: Chronic Kidney Disease, ESRD: End-Stage Renal Disease

This particular case highlights that an insulinoma may manifest with severe or life-threatening symptoms as its initial presentation. Rarely do patients have an initial presentation with a blood sugar low enough to induce seizures [9]. In the context of recurrent hypoglycemia in a non-diabetic patient, it is vital to adhere to a systematic and meticulously planned diagnostic approach (Figure 3). This approach is crucial in excluding the potential presence of an insulin-secreting tumor as various other prevalent conditions, including acute and chronic illnesses, as well as hormonal disorders, can initially exhibit recurrent hypoglycemia in non-diabetic patients. The management strategies for these diverse causes differ significantly. Additionally, a systematic and well-organized diagnostic approach is necessary in order to prevent misdiagnosis which could lead to fatal recurrence of hypoglycemic symptoms if left incorrectly treated. In this patient, it was imperative to conduct a meticulous investigation into the cause of her recurrent hypoglycemia, given that her initial and only symptoms could have potentially resulted in a fatal outcome.

Diagnosis is established through fulfillment of Whipple's triad criteria, which encompass hypoglycemia (plasma glucose <50 mg/dL), neuroglycopenic symptoms, and rapid alleviation of symptoms upon glucose administration. The definitive method for biochemically confirming the diagnosis involves evaluating plasma glucose, insulin, C-peptide, and proinsulin levels in the bloodstream during a 72-hour fasting period (Table 2) [13]. While under normal circumstances, the production of endogenous insulin is suppressed during hypoglycemia, the persistence of insulin release throughout a 72-hour fast strongly indicates the presence of an insulin-secreting tumor. Throughout the test, blood glucose levels are measured using a reflectance meter at intervals of four hours (and hourly when blood glucose falls below 60 mg/dL). If readings dip below 50 mg/dL or if the patient displays hypoglycemic symptoms, a blood sample is collected to assess serum glucose, insulin, proinsulin, and C-peptide concentrations, subsequently concluding the fasting period (Figure 3).

**Table 2:** Hypoglycemia labs in various hypoglycemic conditions. C-peptide = Insulin connecting peptide, NIPHS = Noninsulinoma pancreatogenous hypoglycemia syndrome, BHB = Beta Hydroxybutyrate

Labs under hypoglycemic conditions (Glucose <55mg/dL)						
	Insulinoma	NIPHS	Sulfonylurea	Exogenous Insulin	Insulin Autoimmune Syndrome	Non-Insulin Mediated
C-peptide	High	High	High	Low	High	Low
Proinsulin	High	High	High	Low	High	Low
Insulin	High	High	High	High	High	Low
BHB	Low	Low	Low	Low	Low	Low
Sulfonylurea Screen	Negative	Negative	Positive	Negative	Negative	Negative
Insulin Antibodies	Negative	Negative	Negative	Negative	Positive	Negative

Once the biochemical diagnosis of an insulinoma is established, the actual masses can be identified through several means, both invasive and non-invasive [14]. Computed tomography (CT) of the abdomen with IV contrast is the preferred initial diagnostic test for detecting insulinomas, as it shows greater differentiation than the normal pancreatic tissue during the capillary and arterial phases of the contrast bolus and hypervascularity of the insulinoma [14]. In this patient, CT of the abdomen with IV contrast was non-diagnostic in identifying the location of the tumor. MRI is a viable substitute for CT and has comparable or superior sensitivity and specificity [15]. Transabdominal ultrasound (US) can also be used for visualization, however localization of the insulinoma is poor (9% to 64%) [16].

In situations where small insulinomas are not detected using transabdominal US, abdominal CT, and MRI, invasive procedures may be used to help with localization [14]. Endoscopic ultrasonography has been shown to have a sensitivity of 87% to 92%, as reported, although its efficacy is primarily operator-dependent and location-dependent with lower sensitivity for tumors located in the pancreatic tail or outside of the pancreas [17]. Combining transhepatic portal venous sampling and angiography with selective arterial calcium stimulation using hepatic venous sampling is likely the most sensitive diagnostic technique currently available, with reported sensitivity ranging from 94% to 100% [18]. Additionally, abdominal positron emission tomography (PET) scans can also be used. However, most are first identified by CT or MRI.

Surgical excision is the preferred treatment for symptomatic insulinomas, and is usually curative for most patients. Following surgery, individuals with insulinomas typically experience excellent long-term survival rates [19]. Medical management with octreotide and diazoxide can be considered for recurrent hypoglycemia, in patients with unresectable or metastatic tumors and during the preoperative period due to their suppression of endogenous insulin secretion from pancreatic beta cells [20].

## Conclusions

Insulinomas are a rare entity for which more data is needed. Even though patients tend to experience progressive episodes of hypoglycemia leading to the diagnosis of insulinoma, an initial severe episode can have tragic consequences. Swift identification and diagnosis in these patients is crucial for avoiding repeat hypoglycemic events and rehospitalization. This case underlines how a directed approach to evaluating non-diabetic patients with recurrent hypoglycemia can help detect this rare entity early in its clinical course.
